# Smoking status impacts treatment efficacy in smoke-induced lung inflammation: A pre-clinical study

**DOI:** 10.3389/fphar.2022.971238

**Published:** 2022-09-07

**Authors:** Nadia Milad, Marie Pineault, Félix Tremblay, Joanie Routhier, Ariane Lechasseur, Marie-Josée Beaulieu, Sophie Aubin, Mathieu C. Morissette

**Affiliations:** ^1^ Quebec Heart and Lung Institute, Université Laval, Quebec City, QC, Canada; ^2^ Faculty of Medicine, Université Laval, Quebec City, QC, Canada; ^3^ Department of Medicine, Université Laval, Quebec City, QC, Canada

**Keywords:** cigarette smoking, COPD, biologics, monoclonal antibodies, smoking status, treatment timing

## Abstract

**Rationale:** Smoking status and smoking history remain poorly accounted for as variables that could affect the efficacy of new drugs being tested in chronic obstructive pulmonary disease (COPD) patients. As a proof of concept, we used a pre-clinical model of cigarette smoke (CS) exposure to compare the impact of treatment during active CS exposure or during the cessation period on the anti-inflammatory effects IL-1α signaling blockade.

**Methods:** Mice were exposed to CS for 2 weeks, followed by a 1-week cessation, then acutely re-exposed for 2 days. Mice were treated with an anti-IL-1α antibody either during CS exposure or during cessation and inflammatory outcomes were assessed.

**Results:** We found that mice re-exposed to CS displayed reduced neutrophil counts and cytokine levels in the bronchoalveolar lavage (BAL) compared to mice exposed only acutely. Moreover, we found that treatment with an anti-IL-1α antibody during the initial CS exposure delayed inflammatory processes and interfered with pulmonary adaptation, leading to rebound pulmonary neutrophilia, increased BAL cytokine secretion (CCL2) and upregulated *Mmp12* expression. Conversely, administration of anti-IL-1α during cessation had the opposite effect, improving BAL neutrophilia, decreasing CCL2 levels and reducing *Mmp12* expression.

**Discussion:** These results suggest that pulmonary adaptation to CS exposure dampens inflammation and blocking IL-1α signaling during CS exposure delays the inflammatory response. More importantly, the same treatment administered during cessation hastens the return to pulmonary inflammatory homeostasis, strongly suggesting that smoking status and treatment timing should be considered when testing new biologics in COPD.

## Introduction

Tobacco smoking remains the major risk factor for the development of chronic obstructive pulmonary disease (COPD), a disease marked by chronic airway inflammation as well as lung tissue destruction and remodeling leading to irreversible airflow obstruction, impaired gas exchange, and increased risk of pulmonary infection (Barnes, 2008; Decramer et al., 2012). Despite our growing knowledge of the underlying pathobiology, COPD remains a difficult disease to manage; the main therapeutic approaches still focus on smoking cessation, optimizing bronchodilation, and controlling inflammation with corticosteroids (COPD, 2018). In recent years, clinical trials investigating the efficacy of several monoclonal antibodies and small molecule inhibitors targeting specific inflammatory pathways have been conducted in COPD, unfortunately without significant success.

It has been suggested that smoking status may have an impact on the efficacy of immunomodulatory therapies in development for COPD patients, since these treatments aim to block specific pathways that may play different roles in smokers versus ex-smokers. As shown in Table 1, some clinical trials of biologics and cytokine inhibitors targeting inflammatory pathways in COPD have found differences in therapeutic efficacy when data were stratified by smoking status at treatment initiation. For instance, the CXCR2 antagonist, navarixin, resulted in mild improvements in lung function decline in the whole cohort of COPD patients, mainly by benefiting active smokers with no significant improvement in ex-smokers (Rennard et al., 2015). Conversely, a recent clinical trial of an IL-33 blocking antibody in COPD patients found that the improvements in lung function and exacerbation rate observed in the cohort as a whole were mainly amongst ex-smokers, with no clinical benefit to current smokers (Rabe et al., 2021). Although important differences in efficacy have been observed between smokers and ex-smokers, few preclinical and clinical studies have focussed on the specific impact of smoking status and treatment timing on drug efficacy in models of cigarette smoking and COPD.

**TABLE 1:** Impact of smoking status on the efficacy of biologic treatments and cytokine inhibitors in COPD.

Drug and publication year	Study design	Smoking status	Ex-smoker definition	Effect of smoking status on outcomes	References
ABX-IL8 (anti-IL-8)	Moderate-severe COPD	43 smokers	None	No significant difference in dyspnea score between smokers and ex-smokers	Mahler et al. (2004)
2004	Pilot study	76 ex-smokers			
	5 months				
Infliximab (anti-TNFα)	Moderate-severe COPD	104 smokers	None	No significant difference in CRQ score between smokers and ex-smokers	Rennard et al. (2007); Rennard et al. (2013)
2007/2013	Phase 2	130 ex-smokers			
	44 weeks				
Canakinumab (anti-IL-1β)	Severe COPD	147 smokers/ex-smokers	None	Not determined	Novartis, (2011)
2010	Phase 1/2				
	45 weeks				
Benralizumab (anti-IL-5Rα)	Eosinophilic COPD	38 smokers	None	Not determined	Brightling et al. (2014)
2014	Phase 2a	63 ex-smokers			
	48 weeks				
Navarixin (CXCR2 antagonist)	Moderate-severe COPD	280 smokers	None	Significant improvement in post-bronchodilator FEV_1_ only for smokers	Rennard et al. (2015)
2015	Phase 2	334 ex-smokers			
	6 months				
Mepolizumab (anti-IL-5)	Eosinophilic COPD	411 smokers	Minimum 6 months	Not determined	Pavord et al. (2017)
2017	Phase 3a	1,046 ex-smokers			
	52 weeks	55 nonsmokers			
CNTO6785 (anti-IL-17A)	Moderate-severe COPD	86 smokers	None	No significant difference in pre-bronchodilator FEV_1_ between smokers and ex-smokers	Eich et al. (2017)
2017	Phase 2	101 ex-smokers			
	24 weeks				
MEDI8968 (anti-ILR1)	Moderate-severe COPD	165 smokers	None	Non-significant trend towards decreased acute exacerbation only in smokers	Calverley et al. (2017)
2017	Phase 2	159 ex-smokers			
	52 weeks				
Danirixin (CXCR2 antagonist)	Mild-moderate COPD	235 smokers	Minimum 6 months	No significant difference in ERS score between smokers and ex-smokers	Lazaar et al. (2020)
2020	Phase 2b	389 ex-smokers			
	26 weeks				
Itepekimab (anti-IL-33)	Moderate-severe COPD	156 smokers	None	Significant improvement in exacerbation rate and FEV_1_ only for ex-smokers	Rabe et al. (2021)
2021	Phase 2a	187 ex-smokers			
	24–52 weeks				

Smoker/ex-smoker inclusion criteria: minimum 10 pack-years (except canakinumab, ABX-IL8, and CNTO6785 = 20 + pack-years); COPD, chronic obstructive pulmonary disease, 6 MW: 6-min walk test distance; IL, interleukin; TNF, tumor necrosis factor; CXCR, C-X-C motif chemokine receptor; ERS, evaluating respiratory symptoms COPD; FEV_1_, forced expiratory volume in 1 s; CRQ, chronic respiratory disease questionnaire.

It would be unsurprising that differences exist between smokers and ex-smokers in the pulmonary response to targeted immunomodulatory treatment, since the ongoing pathobiological features exhibited differ significantly (Xu et al., 1994; Scanlon et al., 2000; Bossé et al., 2012; Govindan et al., 2012). The biological processes taking place in the lungs of a heavy smoker (2+ packs per day) compared to those of an individual who quit 5 years ago are not identical, even if they are both diagnosed with same stage of COPD. Though COPD patients who quit smoking exhibit significant improvements in the rate of lung function decline and the risk of acute exacerbation compared to active smokers, ex-smokers still exhibit worsened outcomes compared to patients without a history of tobacco use (Godtfredsen et al., 2002; Hogg et al., 2004; Godtfredsen et al., 2008). Interestingly, pulmonary levels of some genes return rapidly to normal after cessation while others (e.g., AHRR and CYP1B1) remain affected even after more than 25 years of cessation (Bossé et al., 2012). Similarly, in mice exposed to cigarette smoke, several genes are rapidly but transiently upregulated in an attempt to maintain homeostasis following acute exposure; however, longer exposure durations result in a slower return to homeostasis following cessation (Braber et al., 2010; Phillips et al., 2015; Yan et al., 2015; Jubinville et al., 2017). Therefore, the impact of smoking history and treatment timing on therapeutic efficacy may be significant and the need for treatments that can help resolve pulmonary inflammation during smoking cessation would be of great value in COPD patients.

In this study, we sought to explore the impact of smoking status and treatment timing on the inflammatory outcomes associated with cigarette smoke exposure. We hypothesized that the history of cigarette smoke exposure will affect the pulmonary response to future cigarette smoke exposures as well as impact the efficacy of the biologic treatment, anti-IL-1α, in a mouse model of cigarette smoking. Firstly, we found that mice adapt to cigarette smoke exposure during cessation, where animals pre-exposed for 2 weeks followed by a 1-week cessation exhibited reduced pulmonary neutrophil infiltration and BALF cytokine levels in response to an acute 2-day re-exposure compared to mice exposed only for 2 days. Interestingly, administration of anti-IL-1α during the initial cigarette smoke exposure transiently reduced pulmonary neutrophilia but led to a rebound in neutrophil infiltration, cytokine secretion and macrophage *Mmp12* expression following 1 week of cigarette smoke cessation and treatment washout. Conversely, IL-1α blockade during smoking cessation significantly mitigated pulmonary neutrophilia, BAL cytokine levels and macrophage protease expression without rebound effects after antibody washout. In all, it seems that targeted immunomodulation during cigarette smoke exposure could interfere with pulmonary adaptation while treatment during smoking cessation would accelerate the resolution of inflammation.

## Materials and methods

### Animal housing, cigarette smoke exposure, and antibody injection protocols

Female 6- to 8-week-old BALB/c mice were purchased from Charles River Laboratories (Montréal, QC, Canada) and housed following guidelines from the Guide for the Care and Use of Laboratory Animals of the Canadian Council on Animal Care (CCAC) at the Québec Heart Lung Institute. Animal protocols were approved by the Committee on the Ethics of Animal Experiments of Université Laval (#2019-208). Mice were exposed to room air (RA) or cigarette smoke (CS) using a whole-body exposure system (SIU24; Promech Lab AB, Vintrie, Sweden) (Morissette et al., 2015; Jubinville et al., 2017). Mice were exposed for 2 h every morning over 5 days/week for 2 weeks to the mainstream cigarette smoke of 24 cigarettes with filters removed (3R4F reference cigarettes; University of Kentucky, Lexington, KY, United States). After 1 week of smoking cessation, mice were acutely re-exposed 2 h per day for 2 consecutive days. For antibody administration during initial cigarette smoke exposure, 6-to-8-week-old mice of similar body weight (approximately 18–20 g) were given an intraperitoneal injection 1 h prior to daily cigarette smoke exposure containing 200 ug of one of the following antibodies (BioXCell, West Lebanon, NH, United States) diluted in 100 μl PBS: control isotype control antibody (BE0091) or hamster anti-mouse IL-1α (BE0243). For antibody administration during cessation, the same antibodies were injected intraperitoneally at the same daily dose in the morning for 4 consecutive days. All sacrifices were performed the morning after the last day of cigarette smoke exposure or the morning after the last day of cessation.

### Bronchoalveolar lavage cell counts

For the bronchoalveolar lavage (BAL), lungs were removed from the thoracic cavity, the trachea was cannulated, and lungs were lavaged with 2 washes of 500 μl for BAL cell counts and separately lavaged with 3 washes of 1 ml of cold PBS to recover as many pulmonary macrophages as possible for cell culture. Lung total cell count was done in the BAL using a hemocytometer. BAL cells were then centrifuged at 800 g 4°C and the supernatant BAL fluid (BALF) stored at −80°C. After cells were resuspended, cytospins were prepared and stained using the Hema 3 protocol (Thermo Fisher Scientific, Waltham, MA United States) to perform differential cell counts where a total of 300 cells were counted per mouse. Pulmonary macrophage size, which is increased in smokers and preclinical models of cigarette smoke exposure and is associated with impaired macrophage phagocytic ability (Dewhurst et al., 2017), was measured using the ImageJ Software (v1.6; ImageJ, http://imagej.nih.gov/ij) as follows: 30 macrophages per cytospin per mouse were randomly selected using the grid plugin, the surface area was measured and the mean size is expressed as a percentage of the mean macrophage size of room air-exposed, isotype-antibody injected mice (RA-Iso).

### Pulmonary macrophage isolation and *ex vivo* cell culture

Remaining cytospin BAL cells were pooled with the three additional 1 ml lavages, then centrifuged at 800 g 4°C, re-suspended in RPMI (Wisent Bioproducts, St-Bruno, QC, Canada) and macrophages were isolated by adherence to a culture-treated 96-well plate (5.0 × 10^4^ pulmonary macrophages per well; 1 h at 37°C, 5% CO_2_). Cells were washed with PBS and lysed immediately for RNA isolation using the RNeasy Mini Kit (QIAGEN, Toronto, ON, Canada) containing 1% β-mercaptoethanol (Millipore Sigma, Oakville, ON, Canada) following manufacturer’s instructions. Lysates were kept at −80°C until RNA extraction. Separate wells of macrophages were washed with PBS and cultured with RPMI for 24 h at 37°C, 5% CO_2_. Supernatants were then collected and stored at −80°C for ELISA analysis.

### Cytokine ELISA

Mouse DuoSet^®^ ELISA kits (R&D Systems, Minneapolis, MN, United States) were used to measure levels of CCL2 (DY479), IL-1α (DY400), and TNFα (DY410) in the BALF and/or in macrophage supernatants following manufacturer’s instructions.

### RNA extraction and real-time quantitative PCR

RNA from macrophages was extracted using the QIAGEN RNeasy Mini Kit (#74106; QIAGEN, Toronto, ON, Canada) while RNA was extracted from the lungs using Trizol (Fisher Scientific, Pittsburg, PA, United States) and chloroform method, as previously described (Jubinville et al., 2020). cDNA was prepared with the maximum volume of RNA (15 µl) using the iScript cDNA synthesis kit (Bio-Rad, Mississauga, ON, Canada) following manufacturer’s instructions. Gene expression was evaluated *via* qPCR as previously described (Jubinville et al., 2017) with the SsoAdvanced Universal SYBR Green Supermix (Bio-Rad, Mississauga, ON, Canada). Gene-specific primers (IDT, Skokie, IL, United States) were used to amplify *Mmp12* (NM_008610; GCT CCT GCC TCA CAT CAT AC; GGC TTC TCT GCA TCT GTG AA; 59°C), *Ddit3* (NM_007837.4 and NM_001290183.1; TGC AGA TCC TCA TAC CAG GC; CCA GAA TAA CAG CCG GAA CCT; 60°C), *Grp78* (NM_001163434.1; ACT TCA ATG ATG CCC AGC GA; AGC CTT TTC TAC CTC ACG CC; 60°C) and two reference genes: *Rplp0* (NM_0074475.5; ATC ACA GAG CAG GCC CTG CA; CAC CGA GGC AAC AGT TGG GT; 56°C) and *Hprt* (NM_013556; AGC AGG TCA GCA AAG AAC T; CCT CAT GGA CTG ATT ATG GAC A; 57°C). The primers were validated with efficiencies between 90% and 110%. Expression is represented as a fold change of room air-exposed, isotype-treated (RA-Iso) mean expression.

### Statistical analyses

Statistical analyses were performed using Prism 9.3.0 (GraphPad, La Jolla, CA, United States) and the results of multiple group comparisons are indicated with asterisks (*p* < 0.05; one-way ANOVA followed by a Šidák’s multiple comparisons post-test). Where *p*-values are specified in the graph, single comparisons were made using two-tailed, unpaired t-tests (*p* < 0.05).

## Results

### Pulmonary adaptation to cigarette smoke exposure reduces neutrophilia and alters macrophage phenotype

**FIGURE 1:**
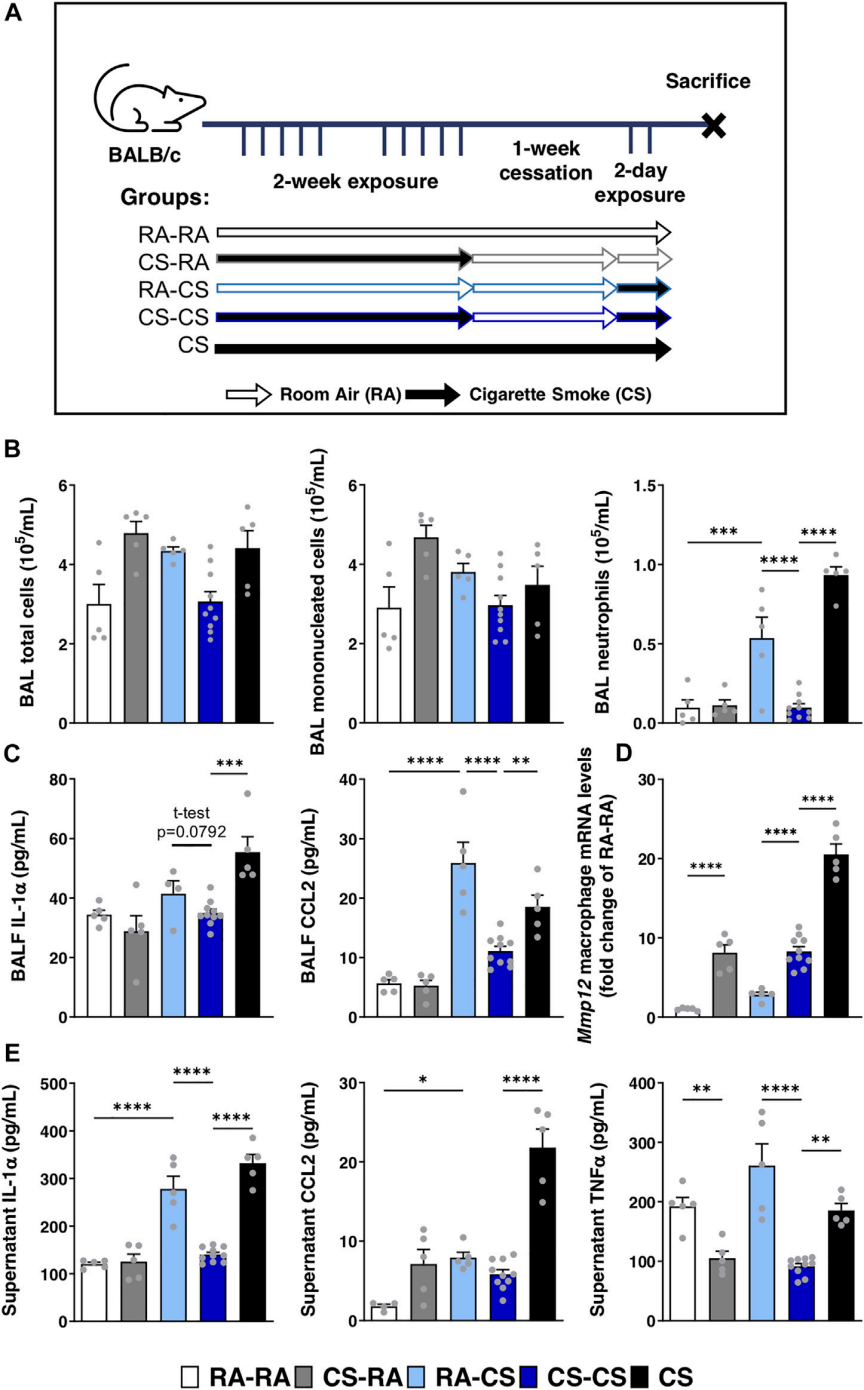
Neutrophilic response to acute cigarette smoke is limited by previous exposures to cigarette smoke. **(A)** Female BALB/c mice were exposed to cigarette smoke 2 h daily 5 days/week for 2 weeks, followed by a 1-week cessation period and finally an acute 2-days exposure. Sacrifices were performed the morning after the final exposure/cessation day. **(B)** Differential cell counts of the BAL were performed. **(C)** Levels of IL-1α, CCL2 and G-CSF in the BALF were measured by ELISA. **(D)** Baseline pulmonary macrophage *Mmp12* mRNA expression levels were quantified by qPCR. **(E)** After 24 h of *ex vivo* BAL macrophage culture, supernatant levels of IL-1α, CCL2 and TNFα were measured via ELISA. Data represented are mean + SEM, *n* = 5–10 mice per group, one-way ANOVA Šidák’s multiple comparisons post-test, except where specified two-tailed t-test *p < 0.05 **p < 0.01 ***p < 0.001 ****p < 0.0001.

Using a model of cigarette smoke exposure, where mice were exposed for 2 weeks, followed by 1-week cessation and acutely re-exposed for 2 days (Figure 1A), we were able to study the pulmonary adaptation to cigarette smoke re-exposure. Despite having been exposed to cigarette smoke for a longer total duration, we found that pre-exposed mice (CS-CS) exhibited reduced neutrophil infiltration in the BAL compared to mice exposed for 2 days only (RA-CS), without affecting total and mononuclear cell counts in the BAL (Figure 1B). This was associated with reduced levels of pro-inflammatory cytokines in the BALF, such as IL-1α (trend, *p* = 0.0792) and CCL2 (Figure 1C). These effects are in contrast with the neutrophilia and elevated cytokine levels observed in the CS group continuously exposed to cigarette smoke throughout the protocol (Figures 1B,C), suggesting that the cessation period is necessary for the development of this pulmonary adaptation.

Pulmonary macrophages isolated from the BAL of these mice were either lysed at baseline for mRNA quantification *via* qPCR or cultured *ex vivo* for 24 h to assess pro-inflammatory cytokine secretion. Interestingly, we found that macrophage expression of *Mmp12* remained elevated in CS-CS mice compared to mice exposed for only 2 days (RA-CS; Figure 1D). Since *Mmp12* expression was similar between CS-RA and CS-CS groups, it suggests that the cessation period was insufficient to renormalize *Mmp12* macrophage mRNA levels upregulated during the initial 2 weeks of exposure (Figure 1D). In line with BALF cytokine levels, *ex vivo* secretion of IL-1α and TNFα by pulmonary macrophages from CS-CS mice was reduced compared to RA-CS mice, without effect on CCL2 secretion (Figure 1E). Taken together, it seems that a pre-exposure to cigarette smoke leads to pulmonary adaptation, which subsequently blunts inflammatory cytokine production by macrophages and reduces neutrophil infiltration following re-exposure, while macrophage expression of *Mmp12* remains elevated.

### Anti-IL-1α treatment during smoke exposure postpones the immune response and interferes with pulmonary adaptation

**FIGURE 2:**
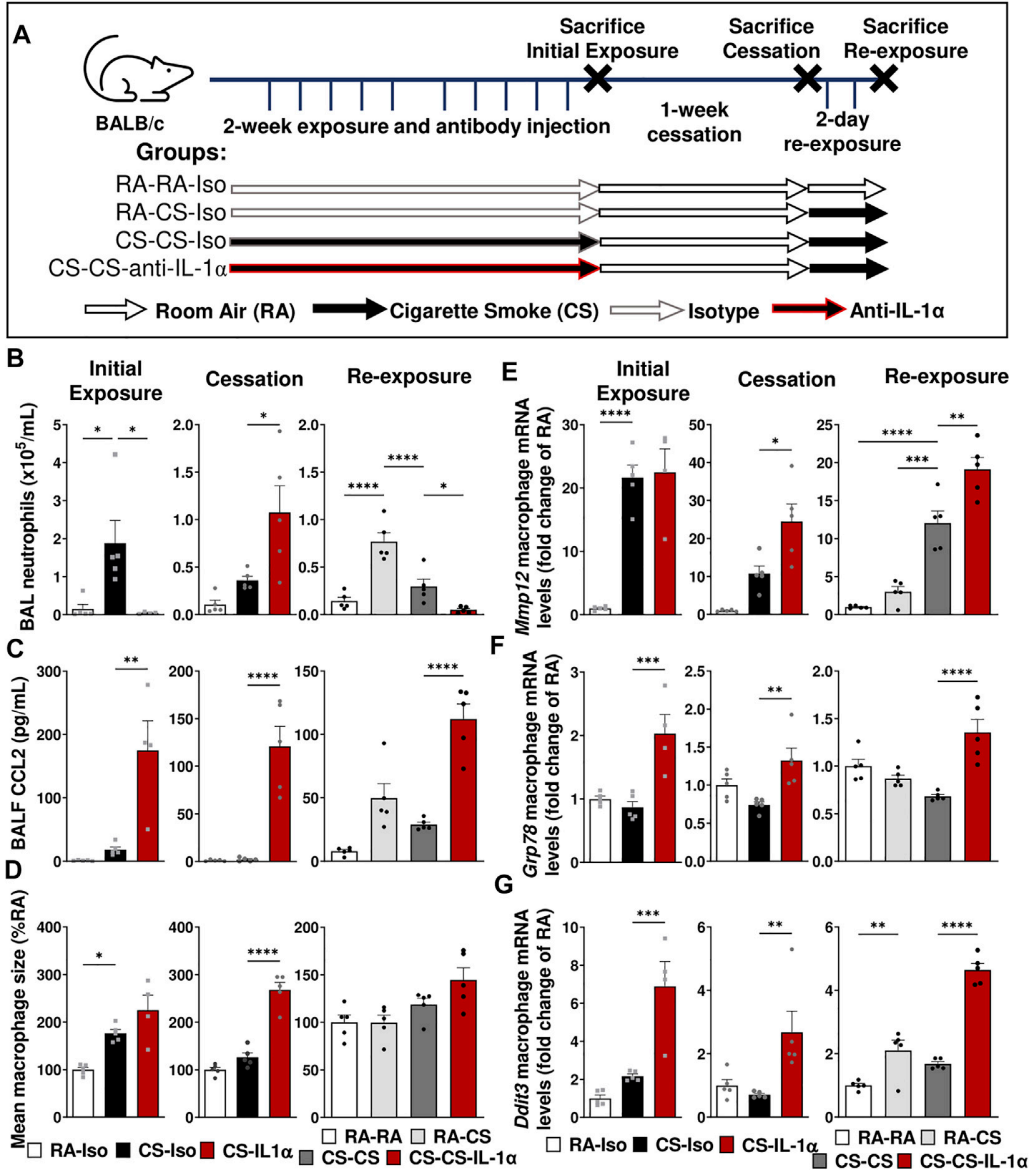
Neutralization of IL-1α during smoke exposure delays the inflammatory response to cigarette smoke exposure and affects pulmonary adaptation. **(A)** Female BALB/c mice were exposed to cigarette smoke 2 h daily 5 days/week for 2 weeks and treated daily with isotype control antibody (Iso) or anti-IL-1α (IL-1α) prior to smoke exposure, followed by a 1-week cessation period and finally an acute 2-day exposure. Animals were euthanized at three timepoints: after the initial exposure and antibody administration (left), following the cessation and antibody washout period (center), and after re-exposure (right). Sacrifices were performed the morning after the final exposure/cessation day. **(B)** BAL neutrophil counts were performed and **(D)** levels of CCL2 in BALF were measured *via* ELISA. **(D)** Pulmonary macrophage size was measured and is expressed as a percentage of the RA-Iso mean. **(E–G)** Baseline pulmonary macrophage mRNA expression levels of *Mmp12*, *Ddit3*, and *Grp78* were quantified by qPCR. Data represented are mean + SEM, *n* = 4–10 mice per group, one-way ANOVA Šidák’s multiple comparisons post-test *p < 0.05 **p < 0.01 ***p < 0.001 ****p < 0.0001.

Next, we sought to assess the impact of blocking IL-1α, a key cytokine involved in the inflammatory response to cigarette smoke, on the pulmonary adaptation to smoke exposure and the return to homeostasis following cessation; in particular, the effects on pulmonary macrophage phenotype and function. To explore this, we administered either isotype control (Iso) or anti-IL-1α (IL-1α) antibodies *via* intraperitoneal injection daily prior to cigarette smoking during the first 2 weeks of exposure. Three timepoints were chosen to explore the impact of treatment at each step (Figure 2A): 1 day after the initial 2-week exposure (left), following 1-week of cessation (center) and 1 day after acute 2-day re-exposure (right).

As expected, after the initial 2 weeks of exposure and antibody administration (Figure 2, left), we found that anti-IL-1α administration during cigarette smoke exposure led to reduced BAL neutrophil counts despite elevated CCL2 levels without effect on BAL macrophage size or *Mmp12* expression (Figures 2B–E). However, following the 1-week cessation and antibody washout (Figure 2, center), we observed a significant rebound increase in BAL neutrophils as well as sustained elevations in BALF CCL2 levels, pulmonary macrophage size (associated with dysregulated macrophage function) and mRNA levels of *Mmp12*, while these parameters returned to room air levels in mice treated with isotype antibody (CS-Iso; Figures 2B–E). Furthermore, genes associated with the endoplasmic reticulum (ER) stress response, such as *Ddit3* (encodes for the chaperone protein CHOP) and *Grp78*, were significantly upregulated in macrophages isolated from CS-IL-1α compared to CS-Iso mice, both immediately after 2 weeks of exposure (left) and after 1 week of cessation and antibody washout (center; Figures 2F,G), indicating an upregulation of the unfolded protein response.

When mice were acutely re-exposed to cigarette smoke after the cessation period (Figure 2, right), mice treated with anti-IL-1α during cigarette smoking (CS-CS-IL-1α) exhibited reduced BAL neutrophil counts compared to similarly exposed mice treated with isotype antibody (CS-CS), while CCL2 levels in the BALF remained elevated (Figures 2B,C). Although macrophage size was not significantly different (Figure 2D), mRNA levels of *Mmp12, Ddit3*, and *Grp78* genes were found to be significantly increased in macrophages isolated from the BAL of CS-CS-IL-1α mice compared to isotype-treated CS-CS animals (right; Figures 2E–G). Taken together, these results indicate that treatment with anti-IL-1α during cigarette smoke exposure affects pulmonary adaptation to cigarette smoke exposure and delays the resolution of pulmonary processes associated with inflammation and tissue destruction.

### IL-1α neutralization during cessation promotes inflammatory resolution and renormalization of pulmonary macrophage phenotype

**FIGURE 3:**
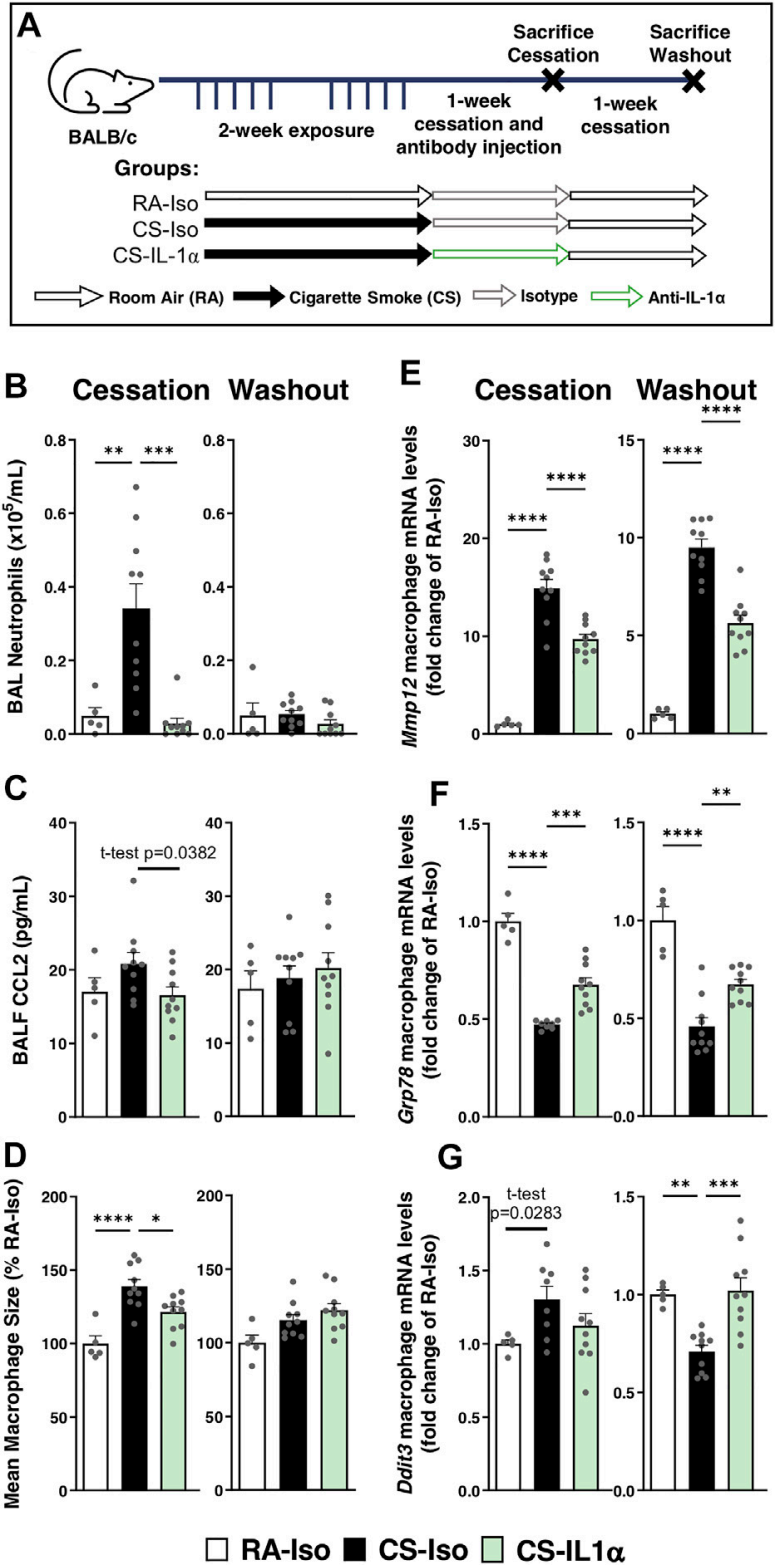
Neutralization of IL-1α during cessation blunts active neutrophil recruitment and hastens inflammatory resolution without rebound effect. **(A)** Female BALB/c mice were exposed to cigarette smoke 2 h daily 5 days/week for 2 weeks and followed by 1-week cessation where mice were intraperitoneally administered isotype control antibody (Iso) or anti-IL-1α (IL-1α) once daily. Mice were euthanized the day after 1 week of cessation and antibody injection (left) or after an additional week of cessation and antibody washout (right). Sacrifices were performed the morning after the final exposure/cessation day. **(B)** BAL neutrophils counts were performed and **(C)** levels of CCL2 in the BALF were measured *via* ELISA. **(D)** Pulmonary macrophage size was measured and is expressed as a percentage of the RA-Iso mean. **(E–G)** Baseline pulmonary macrophage mRNA expression levels of *Mmp12*, *Grp78*, and *Ddit3* was measured via qPCR. Data represented are mean + SEM, *n* = 5–10 mice per group, one-way ANOVA Šidák’s multiple comparisons post-test, except where specified t-test (two-tailed) *p < 0.05 **p < 0.01 ***p < 0.001 ****p < 0.0001.

To determine whether treatment timing has an impact on anti-IL-1α’s efficacy and rebound effects associated with treatment withdrawal, mice were instead treated during the cessation period. As shown in Figure 3A, mice were exposed to cigarette smoke for 2 weeks followed by 1 week of cessation during which they received either isotype or anti-IL-1α antibody injections daily for 1 week. Animals were euthanized after the week of antibody injections (left) or after an additional 1-week cessation period without antibody administration (right) to assess rebound effects after treatment washout (Figure 3A). In contrast to the effects of IL-1α signaling blockade during cigarette smoke exposure, we found that anti-IL-1α treatment during cessation led to a reduction in neutrophil counts in the BAL and mildly decreased BALF levels of CCL2 compared to CS-Iso mice (left; Figures 3B,C). In addition, macrophage size and expression of *Mmp12* were significantly reduced in mice treated during cessation with anti-IL-1α (CS-IL-1α) compared to the isotype-treated group (left; Figures 3D,E). Although ER stress-related mRNA levels of *Grp78* were increased in macrophages from CS-IL-1α mice, mRNA levels remained lower than room air-exposed mice and there was no significant effect on *Ddit3* mRNA levels (left; Figures 3F,G).

To assess the potential effects of treatment withdrawal/washout, we found following 1-week of treatment washout (Figure 3, right) that anti-IL-1α treatment during cessation did not lead to any rebound effects: pulmonary neutrophilia, BALF CCL2 levels, pulmonary macrophage size and *Mmp12* expression remained reduced in CS-IL-1α mice compared to CS-Iso (right; Figures 3B–E). Interestingly, mRNA levels of *Grp78* and *Ddit3* were increased in the CS-IL-1α group compared to isotype-treated animals, though resultant mRNA levels were similar to room air-exposed mice (right; Figures 3F,G). Therefore, smoking status and treatment timing seem to have a dramatic impact on the anti-inflammatory efficacy of anti-IL-1α treatment, as observed by divergent effects on pulmonary neutrophilia, cytokine secretion and macrophage phenotype.

### IL-1α neutralization transiently reduces pulmonary macrophage cytokine secretion *ex vivo*

**FIGURE 4:**
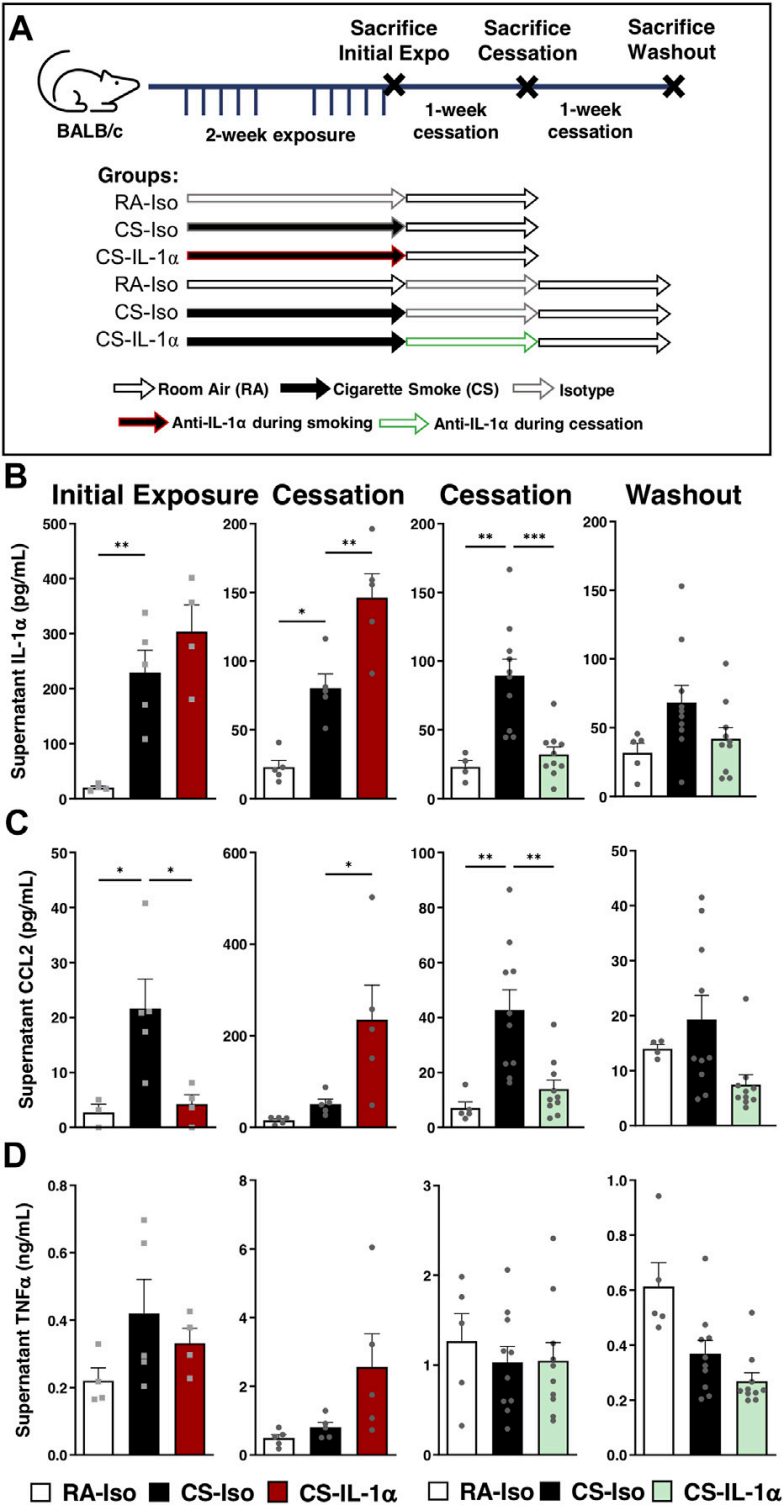
Neutralization of IL-1α *in vivo* during cessation leads to sustained reductions in pulmonary macrophage cytokine secretion *ex vivo*. **(A)** Female BALB/c mice were exposed to cigarette smoke 2 h daily 5 days/week for 2 weeks and followed by 1-week cessation. Mice were intraperitoneally administered anti-IL-1α once daily either during cigarette smoke exposure (left) or during cessation (right) and euthanized after initial exposure (left), following 1-week cessation (center) or after 1 additional week of cessation and antibody washout (right). Sacrifices were performed the morning after the final exposure/cessation day. **(B–D)** Pulmonary macrophage supernatant levels of IL-1α, CCL2, and TNFα were measured via ELISA following 24 h of incubation *ex vivo*. Data represented are mean + SEM, *n* = 4–10 mice per group, one-way ANOVA Šidák’s multiple comparisons post-test *p < 0.05 **p < 0.01 ***p < 0.001.

Since macrophages are the major orchestrators of the inflammatory response to cigarette smoking, we wanted to assess the impact of blocking IL-1α signaling on macrophage function. To do this, we cultured pulmonary macrophages isolated from the BAL of mice treated *in vivo* with anti-IL-1α during smoke exposure or during cessation (Figure 4A) for 24 h, then collected supernatants for ELISA analysis of cytokine levels. As expected, immediately following 2 weeks of cigarette smoke exposure, pulmonary macrophages isolated from CS-Iso mice secrete increased levels of IL-1α and CCL2 compared to room air-exposed animals while levels of TNFα remain unchanged (left; Figures 4B–D). In mice treated with anti-IL-1α during cigarette smoking (Figure 4, left and center left), we observed a transient decrease in pulmonary macrophage CCL2 secretion when cultured *ex vivo* with no effect on IL-1α and TNFα levels (left; Figures 4B–D). However, after 1 week of cessation and antibody washout, we found that anti-IL-1α treatment during exposure led to a significant rebound increase in IL-1α and CCL2 levels in the supernatant without a change in TNFα secretion (center left; Figures 4B–D).

On the other hand, when anti-IL-1α was administered during smoking cessation (Figure 4, center right and right), there was a significant decrease in pulmonary macrophage IL-1α and CCL2 secretion compared to CS-Iso mice, leaving TNFα levels unaffected (center right; Figures 4B–D). Interestingly, secretion of IL-1α and CCL2 did not rebound after an additional 1-week cessation and washout period in CS mice treated during cessation, with CCL2 levels decreased compared to CS-Iso mice (right; Figures 4B–D). Therefore, it seems that blocking IL-1α signaling during cigarette smoke exposure transiently reduces macrophage pro-inflammatory cytokine secretion but leads to a rebound increase upon treatment withdrawal while treatment during cessation leads to sustained reductions in macrophage cytokine production.

## Discussion

**FIGURE 5:**
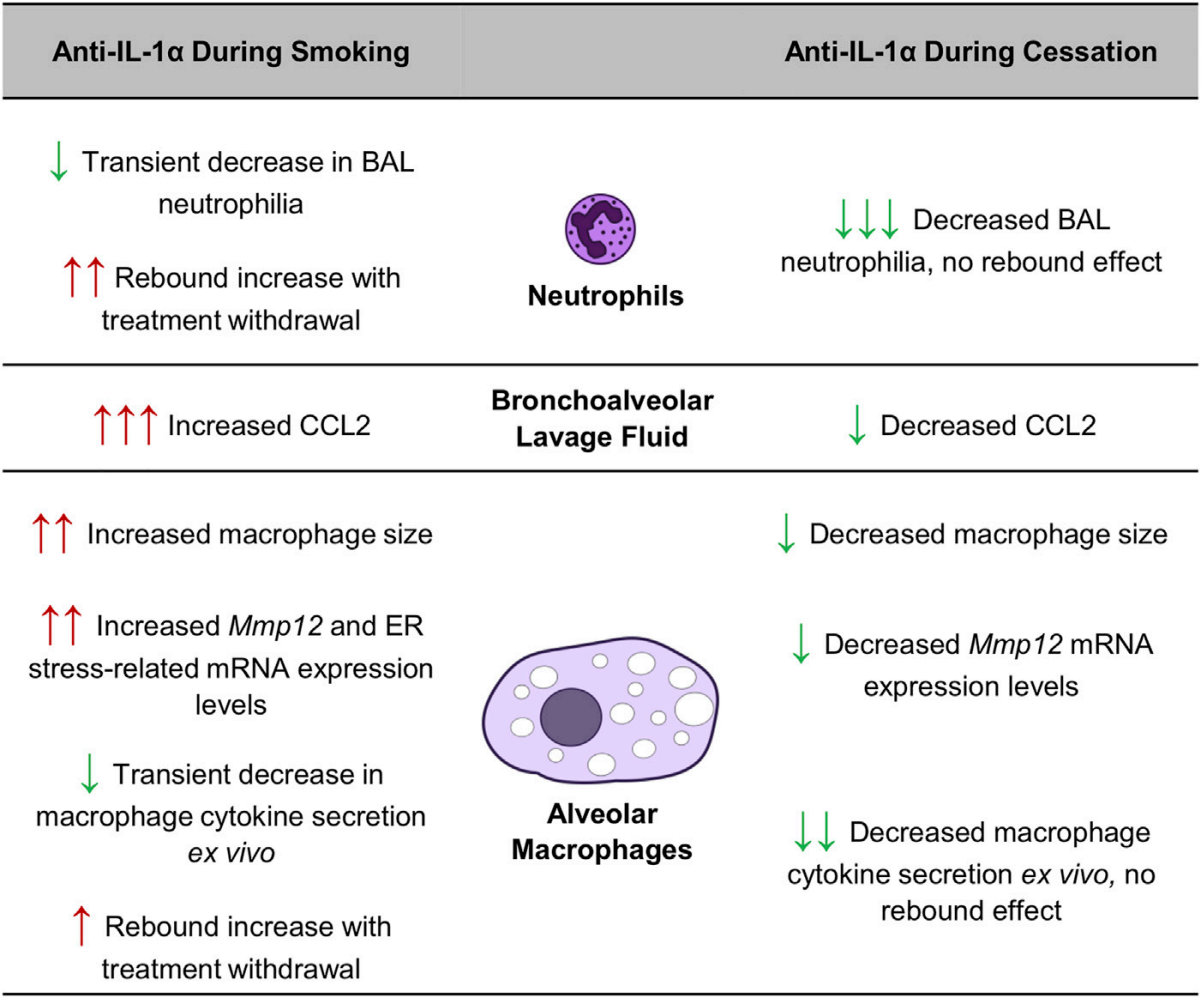
Impact of smoking status and treatment timing on anti-IL-1α treatment efficacy in mice.

In this study, we found that blocking a key immune pathway during cigarette smoke exposure, the IL-1α signaling cascade, can interfere with ongoing adaptive processes, resulting in rebound inflammation upon treatment withdrawal. On the other hand, the same intervention during smoking cessation accelerated inflammatory resolution without causing any rebound effects. These data confirm that treatment timing, recent smoking history and smoking status can have a substantial impact on therapeutic efficacy and outcomes, as summarized in Figure 5. Interestingly, others have observed that repeated exposure to an oxidative stimulus leads to adaptation, showing that three exposures to chlorine gas in mice led to significantly reduced neutrophilia compared to a single exposure, while *Mmp12* expression by pulmonary macrophages was upregulated (Allard et al., 2019). In our study, we found that re-exposure to cigarette smoke following cessation similarly led to reduced pulmonary neutrophilia while macrophage mRNA expression of *Mmp12* remained elevated. Since MMP-12 is a metalloproteinase central to cigarette smoke-induced pulmonary damage (Hautamaki et al., 1997), it seems that although pulmonary adaptation to repeated cigarette smoke exposure mitigates neutrophil infiltration it may not necessarily protect against lung tissue damage in the long term.

Many preclinical studies have explored the inhibition of IL-1 signaling in the context of cigarette smoke exposure and COPD models, where inhibition/deletion of IL-1α or its receptor led to reduced pulmonary neutrophilia and cytokine secretion (Botelho et al., 2011; Morissette et al., 2015; Milad et al., 2021). Positive results from these animal models led to the exploration of IL-1 signaling blockade in COPD patients, though little to no benefit to lung function or exacerbation risk was observed in these trials: e.g., canakinumab and MEDI8968 (Table 1) (Novartis, 2011; Rogliani et al., 2015; Calverley et al., 2017). In our study, although anti-IL-1α administration during cigarette smoke exposure led to an initial improvement in pulmonary neutrophilia, we found that after 1 week of antibody withdrawal and cessation there was a dramatic rebound in neutrophilia, an exaggeration of inflammatory cytokine secretion, an increase in pulmonary macrophage size and an upregulation of the mRNA expression levels of *Mmp12* and ER stress-related genes. Other immunomodulatory treatments have also shown significant rebound effects following treatment cessation. For instance, there is growing evidence that withdrawal of inhaled corticosteroids leads to more rapid lung function decline compared to steroid naïve patients (Burge et al., 2000; Wise et al., 2000; Qaseem et al., 2011). Nevertheless, few preclinical studies have specifically explored treatment during cessation or the potential rebound effects of treatment washout, where the IL-1α/β signaling blockade preclinical studies mentioned above focused on inhibition throughout cigarette smoke exposure (Botelho et al., 2011; Milad et al., 2021).

Furthermore, following re-exposure to cigarette smoke, we observed that anti-IL-1α administration during cigarette smoking disrupted pulmonary adaptation, worsening inflammatory cytokine secretion and affecting pulmonary macrophage phenotype. Along with the observed delayed/rebound inflammation following treatment washout, these data suggest that IL-1α plays a role in the development of pulmonary adaptation to cigarette smoke exposure though the mechanisms that remain unclear. Alveolar macrophages have been repeatedly shown to increase in size and develop a foam cell-like phenotype following cigarette smoke exposure, whether in preclinical or clinical studies (Wilson et al., 2011; Morissette et al., 2015). This has been associated with reduced phagocytic ability and linked to defective lipid export and increased surfactant lipid turnover (Morissette et al., 2015; Dewhurst et al., 2017; Lugg et al., 2021). We found that anti-IL-1α administration during cigarette smoking led to increased mean macrophage size and increase macrophage expression of genes associated with tissue destruction (*Mmp12*) and ER stress (*Grp78* and *Ddit3*). The polarization of pulmonary macrophages towards this destructive and “stressed” phenotype may have important consequences since it has previously been shown that the accumulation of intracellular lipids in large macrophages can lead to the upregulation of ER stress gene expression, leading to macrophage apoptosis and exacerbated cytokine secretion (Yao et al., 2015; Ayaub et al., 2016; Jubinville et al., 2019). This is in line with our findings that anti-IL-1α treatment during cigarette smoking led to increased macrophage expression of *Mmp12* and ER stress genes, increased macrophage size and increased macrophage secretion of IL-1α and CCL2 *ex vivo*. In all, it seems that the inhibition of IL-1α signaling during cigarette smoke exposure delays and exacerbates the inflammatory response.

Conversely, we observed that treatment with the same antibody during the cessation period had the opposite effect, leading to sustained reductions in neutrophilia, BALF CCL2 levels and macrophage metalloproteinase expression, even after treatment withdrawal. In contrast to its inflammation-delaying effects when administered during exposure, anti-IL-1α treatment during smoking cessation seemed to accelerate the resolution of processes associated with lung inflammation and tissue destruction. As summarized in Figure 5, the divergent effects of anti-IL-1α treatment during or after cigarette smoking are striking: IL-1α inhibition during smoke exposure led to increased in neutrophilia following 1-week of cessation while treatment during cessation led to a reduction in BAL neutrophil counts, with no rebound effects following withdrawal. This suggests that IL-1α signaling during cessation in the absence of cigarette smoke stimulus leads to persistent inflammatory signaling and that inhibition of this pathway during cessation can lead to more rapid resolution of inflammation.

These results highlight a novel therapeutic avenue for COPD patients which could be tailored to former smokers as a means of accelerating the restoration of homeostasis and promoting inflammatory resolution during cessation. Although quitting smoking has been shown to slow lung function decline and reduce the risk of exacerbation in COPD patients, ex-smokers with COPD still exhibit increased airway inflammation, lung function decline as well as increased risk of acute exacerbation and death compared to COPD patients without a history of tobacco use (Godtfredsen et al., 2002; Hogg et al., 2004; Godtfredsen et al., 2008). Among the studies of biologic treatments or cytokine inhibitors in COPD where smoking status significantly impacted drug efficacy, most therapies seem to benefit active smokers more than ex-smokers, with the exception of the anti-IL-33 mAb, itepekimab (Table 1) (Rabe et al., 2021). Consequently, the potential to support the resolution of inflammation following cigarette smoke cessation, without merely delaying the response to insult, requires further investigation in the management of COPD patients. Our data also point to the importance of timing treatments to when they would be most effective and when rebound effects would be minimized; although whether drug administration during cessation is ideal for all immunomodulatory therapies remains to be explored. Furthermore, since we focussed primarily on anti-IL-1α’s effects on inflammatory outcomes using an acute model, it would be interesting to assess the impact of treatment timing and withdrawal on lung function decline and the response to infection in long-term models of cigarette smoke exposure, as this would more closely recapitulate COPD pathology.

Overall, the results from our study and from the clinical trials presented in Table 1 highlight the importance of placing recent smoking history and smoking status at the center of clinical trial design in COPD, since the overall lack of efficacy in these trials may in fact be masking divergent effects in active and former smokers. However, due to the current methodology used in preclinical models and in COPD clinical trial design, the impact of smoking status on therapeutic efficacy is extremely challenging to dissect. Smoking status is generally determined only at the first visit without reassessment at follow-up and, as the rate of successful cessation is low (Raherison et al., 2005), this suggests that a fraction ex-smokers relapse during long 6–12-months studies and, vice versa, there is likely a subset of smokers who decided to quit during the trial. Furthermore, insofar as it is stated in the inclusion criteria at all (Table 1), ex-smokers are typically defined as having quit for at least 6 months prior to the beginning of the trial. Though results from these trials are often corrected for smoking history (pack-years), the duration of cessation is not considered, meaning that individuals who quit for over 20 years are pooled with those who quit merely 6 months ago. Therefore, we suggest that future COPD trials should thoroughly document and objectively assess smoking status throughout the trial, as this would allow true assessment of the impact of smoking status and relapse on treatment efficacy.

## Data Availability

The raw data supporting the conclusion of this article will be made available by the authors, without undue reservation.
